# Supplementary material for: Calorie Restriction Suppresses Premature Ageing in Pro-Apoptotic Yeast Mutants Through an Autophagy-Independent Mechanism
Source: Int J Mol Sci. 2026 Jan 1;27(1):464. doi: 10.3390/ijms27010464 (PMC12786662; doi:10.3390/ijms27010464)
Supplement: Supplementary file 1 [file ijms-27-00464-s001.zip › ijms-3995341-supplementary.pdf]

Calorie restriction suppresses premature ageing in pro-apoptotic yeast mutants through an autophagy-independent mechanism

Benedetta Caraba, Mariarita Stirpe, Vanessa Palermo, Alessia Ayala Alban, Arianna Montanari, Michele Maria Bianchi, Claudio Falcone and Cristina Mazzoni

Supplementary material

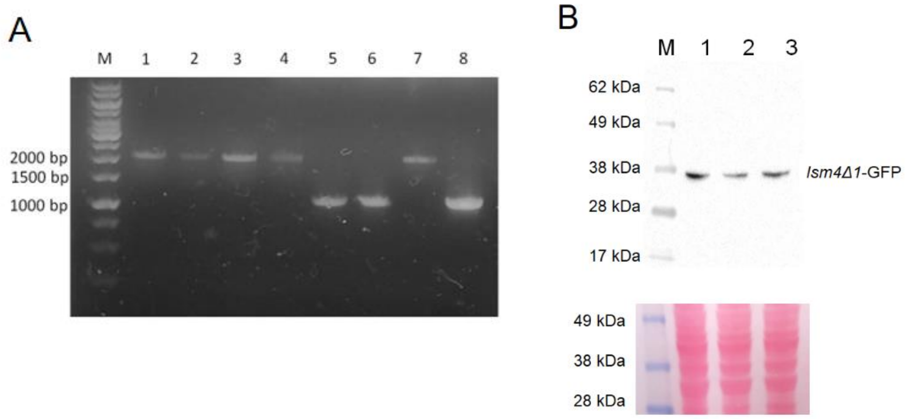

Figure S1. (A) Seven different colonies of BY4741 edited with CRISPR/Cas9 were tested for the integration of the GFP cassette at *LSM4* locus. The PCR fragment in the wild type BY4741 strain (Lane 8) is 1000 bp long, while the edited samples present additional 1000 bp corresponding to the *lsm4Δ1*-GFP cassette, resulting in a 2000 bp band. Five colonies over seven tested result positive to the integration of the cassette, resulting in editing efficiency above 70%. **(B)** Western Blot against GFP of three different colonies of BY4741 *lsm4Δ1*-GFP strain obtained with genome editing. The predicted molecular weight of the fusion protein is 36.4 kDalton and a corresponding band is present in all three samples, confirming the expression of the fusion protein. Ponceau S staining was used as loading control.

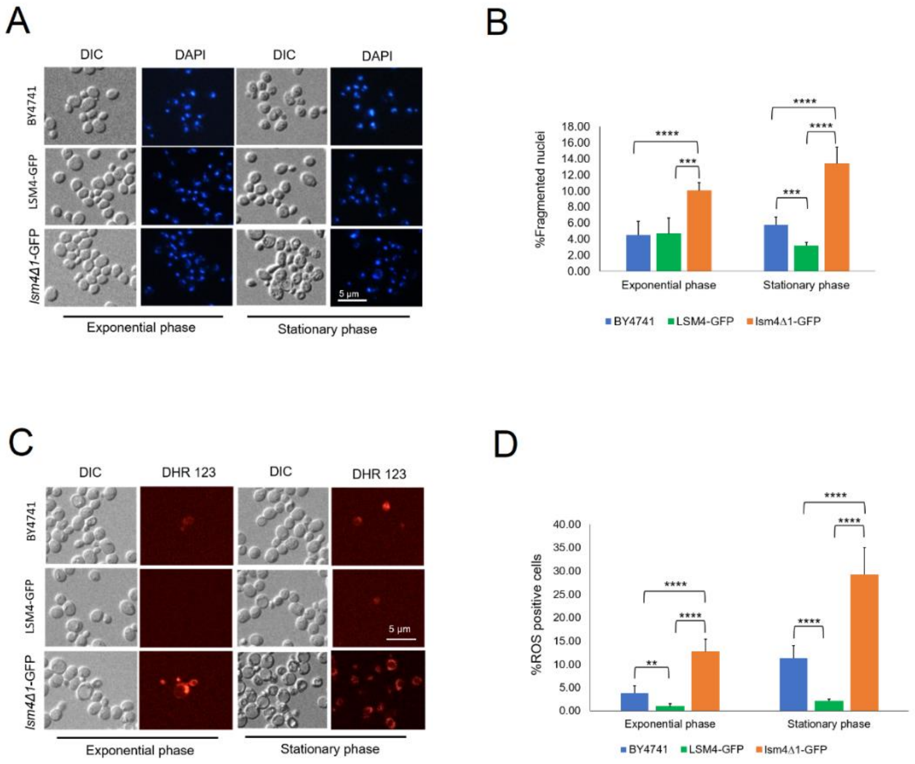

Figure S2. **(A)** DAPI staining of the BY4741, BY4741/*LSM4*-GFP (wild types) and BY4741/*lsm4Δ1*-GFP mutant cells, in both exponential and stationary phase, quantification of the percentage of fragmented nuclei over total cells from three independent experiments is plotted in **(B)**. **(C)** Dihydrorhodamine 123 (DHR-123) staining of the BY4741, BY4741/*LSM4*-GFP (wild types) and BY4741/*lsm4Δ1*-GFP mutant cells in both exponential and stationary phase, quantification of the percentage of ROS positive cells over total cells from three independent experiments is plotted in **(D)**. Data are represented as mean percentage of 700 cells per set ± standard deviation. **pvalue<0.01 ***p-value<0.001, ****p-value<0.0001

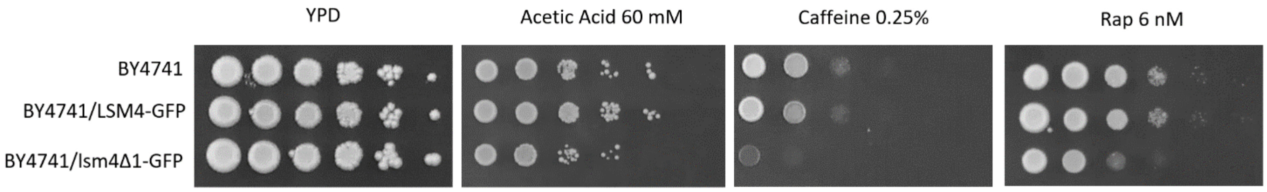

Figure S3. 10-fold dilution of exponential growing cultures in YPD of BY4741 (wild type), BY4741/*LSM4*-GFP and BY4741/*lsm4Δ1*-GFP mutant cells were spotted on complete solid media YPD containing 60 mM acetic acid, 0.25% caffeine and 6 nM rapamycin and plates were incubated at 28°C for 3 days. YPD was used as growth control.

Table S1: OD_{600} values at day 1 referred to the Figure 1

Media	CML39-1A	*Sclsm4Δ1*
SD	2,67 ± 0,12	3,35 ± 0,19
SD 0,1%	2,27 ± 0,04	2,35 ± 0,16
S-glu	0,58 ± 0,06	0,60 ± 0,04
H_2O	1,22 ± 0,05	1,55 ± 0,02
